# Magnetic nozzle radiofrequency plasma thruster approaching twenty percent thruster efficiency

**DOI:** 10.1038/s41598-021-82471-2

**Published:** 2021-02-02

**Authors:** Kazunori Takahashi

**Affiliations:** Department of Electrical Engineering, Tohoku University, Sendai, 980-8579 Japan

**Keywords:** Aerospace engineering, Plasma physics

## Abstract

Development of a magnetic nozzle radiofrequency (rf) plasma thruster has been one of challenging topics in space electric propulsion technologies. The thruster typically consists of an rf plasma source and a magnetic nozzle, where the plasma produced inside the source is transported along the magnetic field and expands in the magnetic nozzle. An imparted thrust is significantly affected by the rf power coupling for the plasma production, the plasma transport, the plasma loss to the wall, and the plasma acceleration process in the magnetic nozzle. The rf power transfer efficiency and the imparted thrust are assessed for two types of rf antennas exciting azimuthal mode number of $m=+1$ and $m=0$, where propellant argon gas is introduced from the upstream of the thruster source tube. The rf power transfer efficiency and the density measured at the radial center for the $m=+1$ mode antenna are higher than those for the $m=0$ mode antenna, while a larger thrust is obtained for the $m=0$ mode antenna. Two-dimensional plume characterization suggests that the lowered performance for the $m=+1$ mode case is due to the plasma production at the radial center, where contribution on a thrust exerted to the magnetic nozzle is weak due to the absence of the radial magnetic field. Subsequently, the configuration is modified so as to introduce the propellant gas near the thruster exit for the $m=0$ mode configuration and the thruster efficiency approaching twenty percent is successfully obtained, being highest to date in the kW-class magnetic nozzle rf plasma thrusters.

Electric propulsion devices in space have recently been recognized as an important technology for space transportation, since their specific impulse is generally larger than that of chemical propulsion devices^1–5^. In the electric propulsion devices, the propellant gas is ionized and energized by coupling an electric power to the ionized gas; resulting in an increase in the specific impulse, which is proportional to an exhaust velocity of the propellant. Some types of the electric propulsion devices, e.g., gridded ion thrusters and Hall effect thrusters, have been successfully developed for the last several decades and practically used in space^6–8^. Electrodes for the ionization (plasma production) and/or for the acceleration of the charged particles have to be exposed to the plasmas in these types of the devices to couple the DC electric power with the plasmas; the electrodes are damaged by ion sputtering and thermal load. Since the device lifetime is limited by the electrode lifetime, its extension has been one of challenging topics in the field of the electric propulsion^9^, especially being important for high power electric propulsion devices. One of candidates overcoming the lifetime problem is an electrodeless plasma thruster having no electrode contacting to the plasmas, where the electric power has to be coupled with the plasma via radiofrequency (rf) or microwave electromagnetic fields^2,5^.

Various types of the electrodeless plasma thrusters have been proposed and investigated so far, e.g., a variable specific impulse plasma rocket (VASIMR)^10^, a helicon double layer thruster (HDLT)^11,12^, a magnetic nozzle rf plasma thruster (often called a helicon thruster: HPT)^13–15^, and an electron cyclotron resonance plasma thruster (ECRT)^16^. These utilize an expanding magnetic field (called a magnetic nozzle) downstream of the plasma source, where various plasma acceleration and momentum conversion processes occur as vigorously investigated so far. In VASIMR, a primary rf power is used to ionize the propellant gas and another rf heating power is coupled with the ions via an ion cyclotron resonance heating process, which increases the ion energy perpendicular to the magnetic field. The perpendicular energy of the ions is converted into the parallel energy in the magnetic nozzle. By increasing the ion heating power up to 200 kW, the thruster efficiency exceeding 50% has been obtained in a laboratory test^10^. On the other hand, the rf power is mainly coupled with electrons and utilized for the plasma production in the HDLT, the HPT, and the ECRT, which are operated at rf power levels ranging from several tens of W to several kW; the electron thermal energy is often converted into the directed ion energy via electrostatic ion accelerations in a current-free double layer and an ambipolar electric field^17^, where the accelerated ions are neutralized by electrons overcoming the potential drop^18^. Fundamental studies on such an electron-heated magnetic nozzle plasma thruster have shown that an internal azimuthal plasma current induces an axial Lorentz force in the magnetic nozzle, resulting in an increase in the thrust^19–21^. The azimuthal plasma current has been identified to be mainly driven by an electron diamagnetic drift, which is induced by a radial pressure gradient^22,23^. Therefore, the radial electron pressure is converted into the axial plasma momentum flux in the magnetic nozzle, where the electron temperature is decreased along the axis by losing their internal energy^24–28^. A two-dimensional magnetic nozzle thruster model in Ref.^29^ can quantitatively explain the measured thrust and can be approximately rewritten by a one-dimensional model using a paraxial approximation, being similar to a physical nozzle model^30^. However, the thrust estimated by the paraxial approximation is a few tens of percent smaller than that by the two-dimensional model. It is expected that the two-dimensional structure of the plasma flow affects the thrust in the magnetic nozzle.

Fundamental laboratory experiments on the rf plasma sources have shown that a peripheral high density region is formed in an inductive mode, while a central density peak is formed simultaneously with an appearance of a helicon wave^2,31^. The excitation of the helicon wave often produces a high density plasma downstream of the source due to the wave propagating in the plasma column^32^. Several experiments in the expanding magnetic fields have shown that the high density conics are formed in the magnetic nozzles^33–36^. Two-dimensional measurement of an electron energy distribution has indicated that the high temperature electrons heated by the rf antenna field are transported along the expanding magnetic field^37^. Therefore, the structure of the electromagnetic fields would play an important role in the formation of the two-dimensional structures, which would affect the imparted thrust as discussed above. Numerical studies have implied that the rf power is absorbed in the magnetic nozzle region downstream of the source due to the wave propagation^38^ as discussed in the experiment^39^. As the wave excitation and the rf power coupling are affected by the antenna structure, an azimuthal mode number *m* of the excitation mode, and the boundary conditions at the walls^40–42^, the optimization of the antenna structure for the thruster configuration is an important experimental issue to be further investigated. The above-mentioned experiments for the plasma source have often been performed with the $m=0$ and $+1$ mode antennas. Investigations on the antenna effect on the thruster performance are required for the performance improvement since the thruster performance is not simply determined by the local plasma density, while different antennas have been tested for the different source geometries and magnetic fields, e.g., in Refs.^12–15^. Therefore, testing different antennas in the unchanged source geometry will provide an insight on the antenna optimization for the thruster performance.

**Figure 1:**
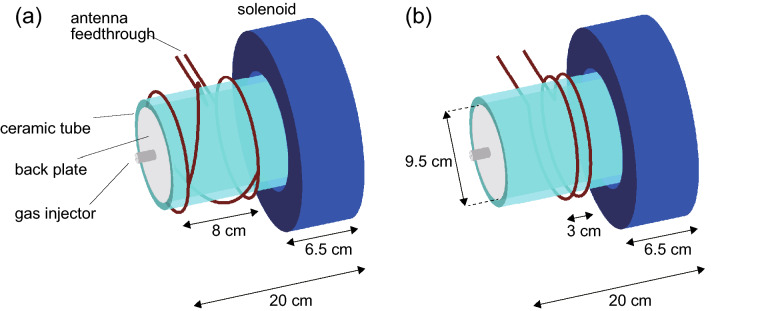
Schematic diagrams of the magnetic nozzle rf plasma thrusters with (**a**) the $m=+1$ mode helical antenna and (**b**) the $m=0$ mode double-turn loop antenna.

In addition to the structure of the electromagnetic fields, analytical, numerical, and experimental studies have shown that a neutral density profile affects both the spatial and temporal behaviors of the plasma density, especially in highly ionized plasmas^43–47^. When the neutral gas is introduced from the upstream side of the source, the neutral density is depleted near the thruster exit due to the high ionization rate and the plasma density is lowered there as predicted in an analytical model^48^ and observed in the experiment^49^. In such a density profile having the axial density peak in the upstream side, a non-negligible axial momentum flux is transferred to the radial wall, corresponding to the loss of the thrust to the wall^50,51^. One of the experiments has shown that the thrust loss can be inhibited by injecting the gas near the thruster exit and that the thrust can be increased by introducing the gas near the thruster exit^52^, in addition to the inhibition of the loss by the magnetic field^53^.

**Figure 2:**
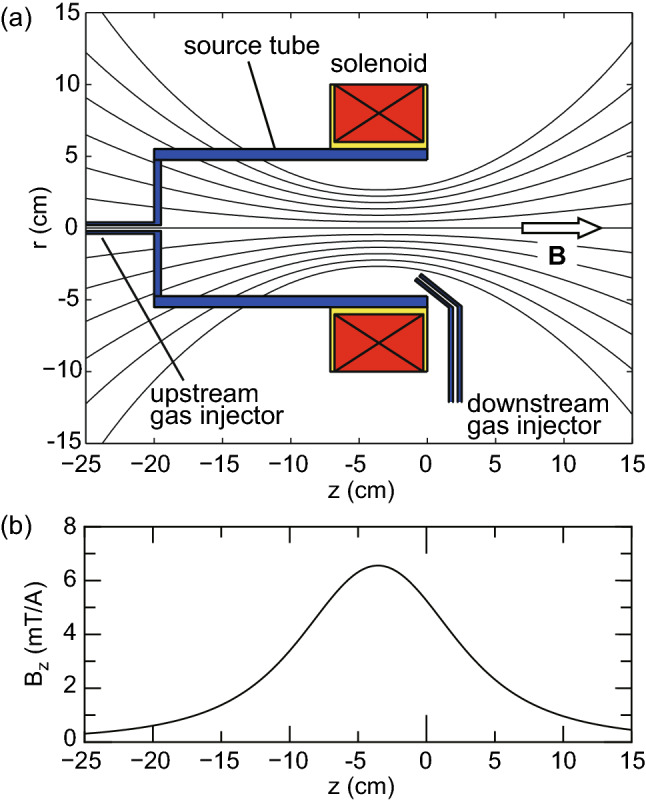
(**a**) Schematic diagrams of the thruster structure consisting of the insulator source tube, the insulator back plate, the upstream and downstream gas injectors, and the solenoid, together with the calculated magnetic field lines (solid lines). A block arrow shows the direction of the magnetic field. (**b**) Calculated magnetic field strength on the *z* axis for the solenoid current of $I_B=1$ A. The magnetic field is convergent near the thruster exit and expanding downstream of the source. Only the field strength can be changed by the solenoid current $I_B$ while maintaining the structure of the field lines.

Several experiments have been performed to improve the performance of the magnetic nozzle rf plasma thruster, where the first direct thrust measurement showed the thruster efficiency less than a percent^54,55^. The subsequent experiment has shown that the thrust due to the magnetic nozzle can be increased by increasing the magnetic field, where the thrust approaches the theoretical limit assuming no plasma loss from the magnetic nozzle^22^. The upper limit of the thrust can be increased by enlarging the diameter of the source tube as predicted by combining the magnetic nozzle model with the global plasma production model^56^. The previously reported thruster efficiency calculated with the rf power is at most $\sim \, 10\%$ for an rf power level of several kW^56^.

Here the investigation on the effect of the antenna structure is initiated from the comparison between the often-used two modes of $m=0$ and $m=+1$. In the present experiment, two different antennas exciting $m=+1$ and $m=0$ modes shown in Fig. 1 are tested for the unchanged source tube in the magnetic nozzle rf plasma thruster which has the upstream gas injection port and is operated at the rf power of several kW, where the rf power transfer efficiency, the plasma density at the radial center, and the imparted thrust are compared between the two cases. A better power coupling and a higher plasma density at the radial center near the thruster exit are obtained for the $m=+1$ mode antenna, while an imparted thrust for the $m=0$ mode antenna is larger than that for the $m=+1$ mode antenna. The configuration is subsequently modified so as to introduce the argon gas near the thruster exit for the $m=0$ mode; the thruster efficiency estimated from the thrust, the mass flow rate, and the rf power, approaches twenty percent, being the highest efficiency to date in the magnetic nozzle rf plasma thruster operated with the rf power of the several kW level. The highest performance is resultantly originated from combining the insights of the effectiveness of the $m=0$ mode antenna, the large diameter source tube, the sufficient magnetic field strength, and the gas injection near the thruster exit.

## Experimental setup

Experiments are performed in a 1-m-diameter and 2-m-long cylindrical vacuum chamber, details of which are described in “Method” section. A pendulum thrust balance is installed inside the vacuum chamber and a thruster structure shown in Fig. 2a is attached to the balance. The thruster consists of a 9.5-cm-inner-diameter, 11-cm-outer-diameter, and 20-cm-long ceramic source tube and a solenoid located near the open source exit. The upstream side of the source tube is terminated by an insulator plate having a small center hole. Two 2-mm-outer-diameter and 1-mm-inner-diameter ceramic tubes are set both at the center hole of the back plate (an upstream gas injector) and near the open source exit (a downstream gas injector) for upstream and downstream gas injections. Argon gas is continuously introduced via either the upstream or downstream gas injector, and the gas flow rate is maintained at 70 sccm (2.1 mg/s) by using a mass flow controller (with an accuracy of $\pm 1$ sccm), resulting in a chamber pressure of about 25 mPa. By supplying a DC solenoid current $I_B$ to the solenoid, the magnetic field converging near the source exit and expanding downstream of the source is formed as shown in Fig. 2, where the direction of the magnetic field at the radial center is indicated by a block arrow in Fig. 2a. Either the $m=+1$ mode helical antenna (Fig. 1a) or the $m=0$ mode loop antenna (Fig. 1b) is wound around the source tube. The antenna is powered by a 13.56 MHz rf generator via an impedance matching box located outside of the chamber; the introduced argon gas is ionized, and the plasma is produced inside the source tube. Two capacitors in the matching box are tuned to minimize the reflected power and the power reflection is undetectable (less than 1 W) for all the conditions in the present experiment.

The rf power transfer efficiency $\eta _p$ and the local plasma density $n_p$ are estimated by measuring the antenna current and by using a Langmuir probe. The equilibrium position of the pendulum balance is displaced when the plasma imparts a force to the thruster; the displacement induced only by the plasma production is measured by using a precise laser displacement sensor (with a resolution of $0.1\,\upmu \mathrm{m}$). The absolute value of the force corresponding to the thrust *F* can be obtained by multiplying a calibration coefficient relating the displacement to the force. More detailed setup and experimental procedures can be found in “Method” section.

## Results

**Figure 3:**
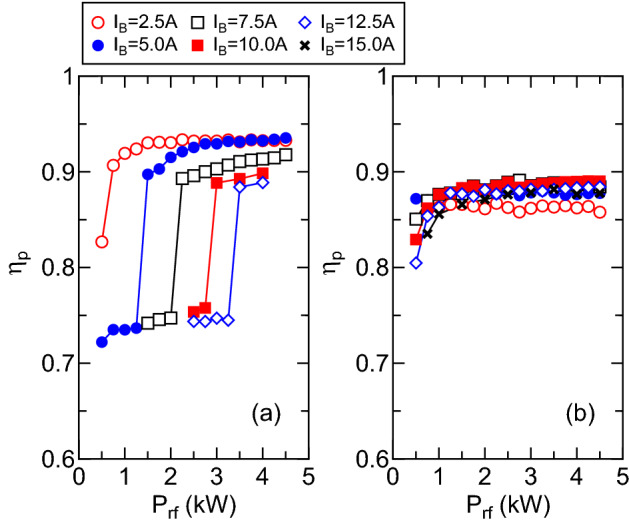
Measured rf power transfer efficiency $\eta _p$ for various values of the solenoid current $I_B$ as a function of the rf power $P_{rf}$ for (**a**) the $m=+1$ mode helical antenna and (**b**) the $m=0$ mode loop antenna. Typical error bar is about $\pm \,5\%$. The discontinuous change in $\eta _p$, which would be a discharge mode transition, is observed for the $m=+1$ case and the efficiency of $\eta _p>0.9$ is obtained for the efficient coupling conditions, while the efficiency for the $m=0$ case is less than 0.9 with no mode transition.

Figure 3 shows the measured rf power transfer efficiency $\eta _p$ for various values of the solenoid current $I_B$ as a function of the rf power $P_{rf}$, where the data in Fig. 3a,b are taken with the $m=+1$ and $m=0$ mode antennas, respectively, and the upstream gas injector is used. As clearly seen in Fig. 3a, the power transfer efficiency $\eta _p$ discontinuously increases when increasing the power; the discontinuous change is considered to be the mode change from capacitive- to inductive- or wave-coupled modes. The rf power threshold of the transition from the low to high efficiencies is found to increase with an increase in the magnetic field strength. The similar feature has been observed in the helicon source experiments, e.g., in Ref.^57^. After the transition to the efficient coupling mode, which is probably the helicon wave discharge mode, the power transfer efficiency greater than 0.9 is obtained for all the magnetic field strength. On the other hand, the transition of the discharge mode is not observed, and the power transfer efficiency is nearly constant at about 0.85-0.9 for the $m=0$ mode case as seen in Fig. 3b, which is lower than that for the $m=+1$ mode case.

**Figure 4:**
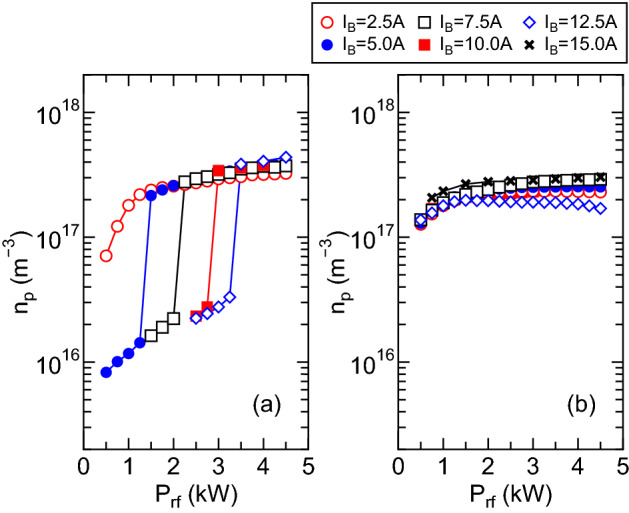
Plasma density measured on the *z* axis at $z=0$ (open source exit) for various values of the solenoid current $I_B$ as a function of the rf power $P_{rf}$ for the (**a**) $m=+1$ and (**b**) $m=0$ mode antennas. Typical error bar in the measurement is about $\pm \,5\%$. The discontinuous change in the density is seen for the $m=+1$ case simultaneously with the transition in the power transfer efficiency (see Fig. 3a), while no discontinuous change is detected for the $m=0$ mode case.

The plasma density $n_p$ as a function of the rf power $P_{rf}$ for the upstream gas injection is measured at the open source exit and the radial center, i.e., $(z, r)=(0, 0)$, for the $m=+1$ and $m=0$ mode cases, as plotted in Fig. 4a,b, respectively. Simultaneously with the discontinuous change in the power transfer efficiency (see Fig. 3a), the density jump by an order of magnitude is detected as shown in Fig. 4a for the $m=+1$ mode case, where the maximum density is about $4\times 10^{17}\,\hbox {cm}^{-3}$. For the $m=0$ mode case, no discontinuous change in the density is detected as well as the rf power transfer efficiency, where the maximum density at $(z, r) = (0, 0)$ is about $3\times 10^{17}\,\hbox {cm}^{-3}$ and slightly lower than that for the $m=+1$ mode.

**Figure 5:**
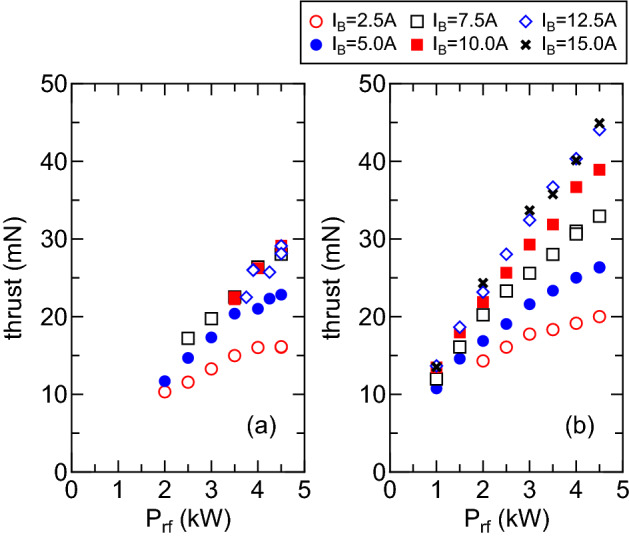
Measured thrust as a function of the rf power $P_{rf}$ for the (**a**) $m=+1$ and (**b**) $m=0$ mode antennas, where the thrust for the low power transfer efficiency conditions is much smaller than that for the high power transfer efficiency mode for the $m=+1$ mode case. Therefore, only the thrust with the high-power transfer efficiency condition are taken in the $m=+1$ mode case. Typical error bar estimated from several shot is about $\pm 3$–$5\%$.

For the same conditions as in Figs. 3 and 4, the thrust is measured by using the pendulum thrust balance for the $m=+1$ and $m=0$ mode cases as shown in Fig. 5a,b, respectively, where the thrust for the low power transfer efficiency conditions in the $m=+1$ mode is very small due to the poor power coupling (Fig. 3) and the low plasma density (Fig. 4); hence only the data with the high power transfer efficiency mode are taken as in Fig. 5a. Both the data in Fig. 5a,b show that the thrust increases with an increase in the rf power $P_{rf}$. As already described, the better power transfer efficiency is obtained for the $m=+1$ mode antenna; nevertheless the thrust for the $m=0$ mode case (Fig. 5b) is significantly larger than that for the $m=+1$ mode case (Fig. 5a).

According to the simple two-dimensional model^22^, the thrust *F* imparted by the magnetic nozzle rf plasma thruster is given by

1 $$\begin{aligned} F = 2\pi \int r p_{e0} dr - 2\pi \int \int r \frac{B_r}{B_z} \frac{\partial p_e}{\partial r} dr dz, \end{aligned}$$

where $p_{e0}$, $B_r$, $B_z$, and $p_e$ are the maximum electron pressure in the source, the radial magnetic field, the axial magnetic field, and the electron pressure, respectively. In Eq. (1), the axial momentum flux lost to the radial source wall is neglected for simplicity, and the axisymmetric system is assumed. The first and second terms in the right-hand side (RHS) of Eq. (1) are the electron pressure force exerted to the back plate, and the Lorentz force arising from the azimuthal electron diamagnetic drift current and the radial magnetic field, respectively. Equation (1) indicates that the thrust could be affected by the two-dimensional profile of the electron pressure.

**Figure 6:**
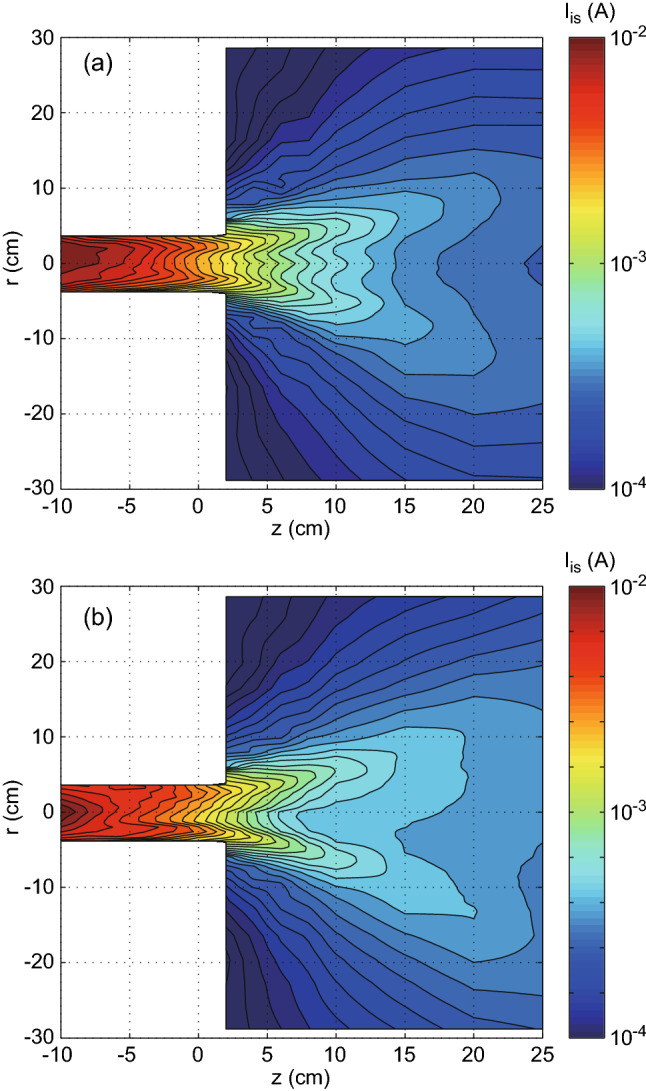
Two-dimensional profiles of the ion saturation current $I_{is}$ of the Langmuir probe taken at the rf power of $P_{rf}=4$ kW for the (**a**) $m=+1$ and (**b**) $m=0$ mode cases. For both the cases, the plasma expands in the magnetic nozzle and the density decay along the axis can be seen, where the density decay along the axis for the $m=+1$ mode case seems to be conspicuous, compared with the $m=0$ mode case.

Previous experiments have shown that the electron temperature is not uniform in the magnetically expanding rf plasmas; the precise identification of the electron pressure profile requires both the density and the electron temperature profiles as measured before^37^; the full sweep of the I–V characteristics of the Langmuir probe have to be taken in the *r*-*z* plane. However it can be very qualitatively characterized by the ion saturation current $I_{is}$ of the Langmuir probe, since $I_{is}$ and $p_e$ are proportional to $n_p \sqrt{T_e}$ and $n_p T_e$, respectively. Figure 6 shows the two-dimensional profiles of the ion saturation current $I_{is}$ of the Langmuir probe taken at the rf power of $P_{rf}=4$ kW for the (a) $m=+1$ and (b) $m=0$ mode cases. The contour lines in the magnetic nozzle region shows that the density or pressure decay along the axis for the $m=+1$ mode case (Fig. 6a) is conspicuous, compared with that for the $m=0$ mode case, which qualitatively implies that the second term in Eq. (1) for the $m=0$ mode case is larger than that for the $m=+1$ mode case.

**Figure 7:**
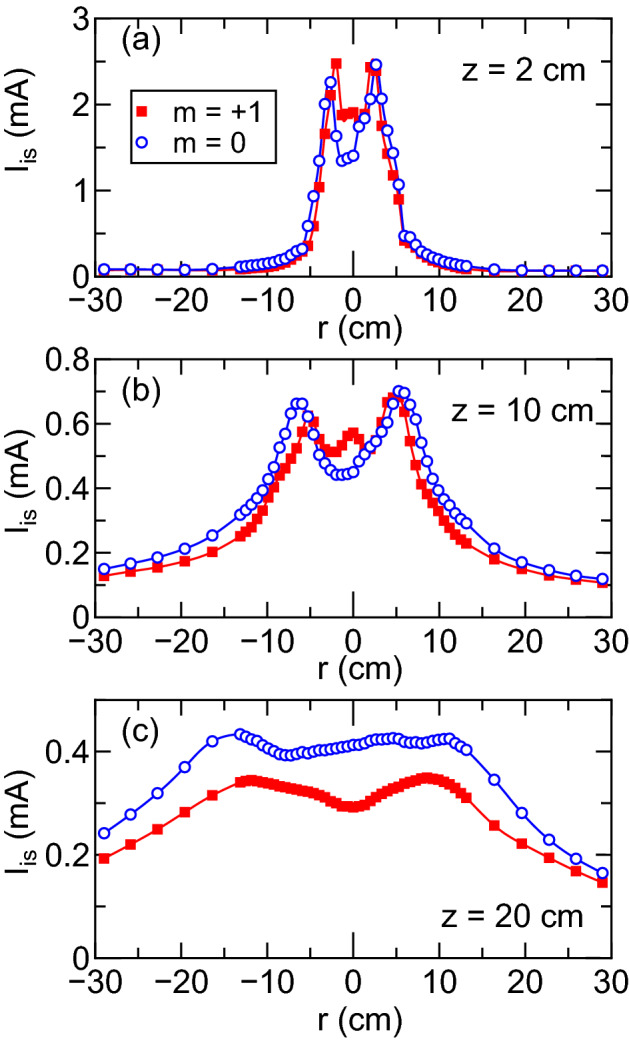
Radial profiles of the ion saturation current $I_{is}$ for the $m=+1$ mode helical (filled squares) and $m=0$ mode loop (open circles) antennas at (**a**) $z=2$ cm, (**b**) $z=10$ cm, and (**c**) $z=20$ cm, where the data are extracted from Fig. 6. The profile for the $m=+1$ mode antenna in (**b**) shows the center peak of $I_{is}$ in addition to the peripheral peak. The measured profiles imply that the plasma radius for the $m=0$ mode case is slightly larger than that for the $m=+1$ mode case. Furthermore, the values of $I_{is}$ for the $m=0$ mode case at $z=20$ cm is about $30\%$ larger than that for the $m=+1$ mode case as seen in (**c**).

**Figure 8:**
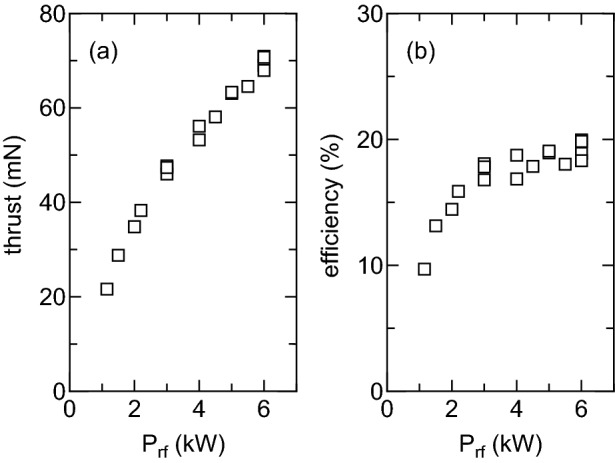
(**a**) Measured thrust as a function of the rf power $P_{rf}$ for the solenoid current of $I_B=15$ A, the gas flow rate of 70 sccm, and the $m=0$ mode antenna configuration, where the argon propellant is introduced from the downstream gas injector. (**b**) The thruster efficiency calculated from the measured thrust in (**a**), the rf power $P_{rf}$, and the mass flow rate of the propellant (2.1 mg/s). The thruster efficiency approaching 20% obtained.

To discuss in more detail, radial profiles of the ion saturation current $I_{is}$ at representative axial positions are shown in Fig. 7. For both the $m=+1$ and $m=0$ mode cases, the peaks of $I_{is}$ are observed at the radially peripheral region of the plasma column, e.g., around $r\sim \pm 2.5$ cm at $z=2$ cm and $r \sim 6$ cm at $z=10$ cm as seen in Fig. 7a,b, respectively. Previous experiments have already shown that the peripheral high density and/or electron temperature region is originated from the electrons heated by the rf antenna and transported along the expanding magnetic field lines^37^. Recent spatial measurement of the plasma momentum flux has also shown that the peripheral high density and temperature region significantly contributes to the thrust generation in the magnetic nozzle^58^. Furthermore, the plasma radius for the $m=0$ mode case seems to be slightly larger than that for the $m=+1$ mode case, which would contribute to the increase in the thrust by the magnetic nozzle according to Eq. (1). For the $m=+1$ mode case, another peak of $I_{is}$ at the radial center can be observed as in Fig. 7b, which is probably due to the excitation of the helicon wave and the resultant ionization at the radial center as observed by Degeling et al. before^31^. However, the contribution of the high density plasma at the central region on the magnetic nozzle thrust term [the second term of the RHS in Eq. (1)] would be small due to the absence of $B_r$ at the radial center. Moreover, $I_{is}$ for the $m=0$ mode at $z=20$ cm is about $30\%$ larger than that for the $m=+1$ mode case as seen in Fig. 7c, despite the similar values of $I_{is}$ for the two antenna cases at $z=10$ cm (see Fig. 7b). Although the mechanisms of the larger value of $I_{is}$ in Fig. 7c for the $m=0$ mode case is still unclear, a possible reason would be a cross-field transport in the magnetic nozzle, which would be significantly affected by the pressure gradient and plasma instabilities. Summarizing the results in Figs. 3, 4, 5, 6 and 7, the large thrust can be obtained for the $m=0$ mode antenna despite the poorer rf power transfer efficiency, compared with the $m=+1$ mode case, due to the larger contribution of the Lorentz force arising from the electron diamagnetic drift current and the radial magnetic field.

The previous experiment has shown that the non-negligible thrust is lost to the radial wall when the propellant gas injected from the upstream side is highly ionized and the neutrals are depleted near the thruster exit^50^. Another experiment using a smaller diameter source tube than the present experiment has demonstrated that the thrust loss is inhibited by injecting the gas near the thruster exit^52^. For the $m=0$ mode antenna configuration, which gives the better performance than the $m=+1$ mode antenna, the argon gas is introduced near the thruster exit by using the downstream gas injector as seen in Fig. 2a, where the gas flow rate is maintained at 70 sccm. Figure 8a shows the measured thrust as a function of the rf power $P_{rf}$ for the solenoid current of $I_B=15$ A and the large thrust is clearly obtained compared with the data in Fig. 5b, e.g., the thrust for the rf power of $P_{rf} \sim 4$ kW and the solenoid current of $I_B=15$ A is $\sim 40$ mN and $\sim 55$ mN in Figs. 5b and 8a, respectively.

The thruster efficiency $\eta$ defined by

2 $$\begin{aligned} \eta = \frac{F^2}{2 \dot{m} P_{rf}}, \end{aligned}$$

is used to assess the thruster performance, where *F*, $\dot{m}$, $P_{rf}$ are the thrust, the mass flow rate of the propellant, and the rf power. It should be noted that the rf power $P_{rf}$ is the output power from the rf generator; hence the dc-rf conversion efficiency in the generator and the electric power for the solenoid are not taken into account here. The thruster efficiency $\eta$ calculated from the data in Fig. 8a is plotted in Fig. 8b; the thruster efficiency increases with an increase in the rf power and approaches about twenty percent, which is the highest values to data as far as the author knows. The presently described performance improvement also contributes to an active space debris removal technology by using the magnetic nozzle plasma thruster^59^. It should be noted that the power consumed for the solenoid is about 900 W, being $15\%$ of the maximum rf power of 6 kW. Therefore, the power consumption for the solenoid would effectively lower the thruster efficiency by a factor of $1/1.15 \sim 0.87$. The influence of the power loss at the solenoid would be lowered when operating the thruster at higher rf power level. Furthermore, replacement of the solenoid by permanent magnets suggested previously^60–62^ will eliminate the power consumption by the solenoid, which is a further challenge for applying the sufficient magnetic field strength to the large diameter source tube.

## Conclusion

Performance of the magnetic nozzle rf plasma thruster is assessed for the two different rf antennas exciting the $m=+1$ and $m=0$ mode electromagnetic fields, where the argon gas is introduced from the upstream side of the source tube. The larger thrust is obtained for the $m=0$ mode antenna configuration due to the larger contribution of the diamagnetic thrust in the magnetic nozzle. For the $m=0$ mode antenna configuration, the thruster efficiency approaching $20\%$ is obtained by injecting the propellant gas near the thruster exit. The estimated thruster efficiency is highest to date in the magnetic nozzle rf plasma thrusters operated at the rf power of the several kW level.

## Methods

### Vacuum chamber and thruster settings

**Figure 9:**
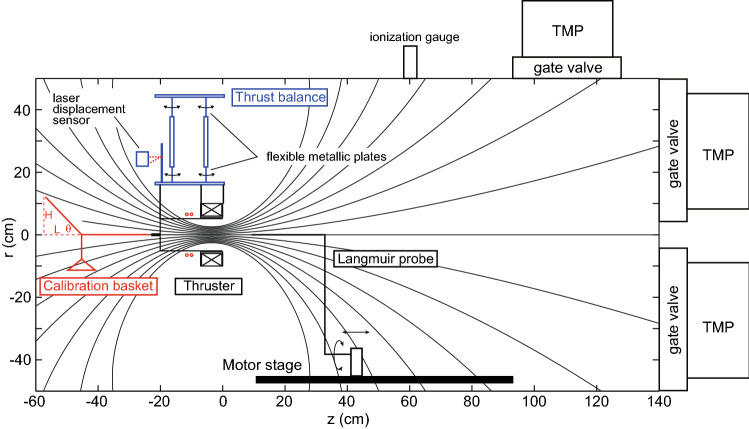
Schematic diagram of the experimental setup including the vacuum chamber, the thrust balance, the thruster, and the Langmuir probe.

Figure 9 shows the schematic diagram of the experimental setup. The 1-m-diameter and 2-m-long vacuum chamber is evacuated by three $3000\,\hbox {Ls}^{-1}$ turbomolecular pumps via gate valves to a base pressure less than $10^{-4}$ Pa, where the effective total pumping speed for argon is about $4500\,\hbox {Ls}^{-1}$. The pendulum thrust balance is installed inside the vacuum chamber^63^, where a bottom aluminum plate is suspended from a top plate attached to the chamber by axially flexible metallic plates, allowing the bottom plate to be displaced by an axial force. The thruster structure including the source tube, the solenoid, and the back plate, is attached to the bottom plate. The rf antenna is wound around the source tube with no mechanical contact to ensure the pendulum motion of the balance. The antenna, which is made of a water-cooled copper tube, is covered by a thick insulator structure and is further shielded by a grounded metallic structure to suppress parasitic discharges outside the source tube^64^ for both the $m=+1$ and $m=0$ mode antennas. Argon gas is continuously introduced from either the upstream or downstream gas injector via a mass flow controller (with an accuracy of $\pm 1$ sccm), and the gas pressure is measured by using an ionization gauge connected to the chamber side port at $z \sim 60$ cm, where the calibration coefficient for argon is used to obtain the absolute value of the pressure. The measured gas pressure is unchanged by the gas injection port and is about 25 mPa for 70 sccm argon gas flow rate. After introducing the gas, the DC solenoid current is turned on; further the rf power is fed to the antenna via a vacuum feedthrough and the impedance matching box; the plasma is produced inside the source tube and expands into the vacuum chamber.

It is well known that the pressure inside the vacuum chamber and the resultant plasma behavior are significantly changed by the pumping speed. Previous experiment with the same vacuum chamber as the present experiment has tested the thrust measurement for different pumping speeds by closing two of three gate valves. As reported in^65^, no change in the thrust by the pumping speed has been detected for the gas flow rate similar to the present experiment.

### Thrust measurement

The axial displacement induced by the plasma production, i.e., the thrust, is measured by a laser displacement sensor (with a resolution of $0.1\,\upmu \mathrm{m}$). For the thrust assessment, the gas is continuously introduced to the thruster the chamber pressure reaches the steady state. The DC solenoid current is supplied; then it is observed that the equilibrium position of the pendulum is moved due to a magnetic force. At about 5–10 s after turning on the solenoid current, the rf power is turned on for about 10 s; then the equilibrium position is further moved due to the force exerted to the thruster by the plasma. From the difference in the equilibrium position with only the solenoid current and with both the solenoid current and the rf power, the displacement induced by the plasma can be obtained. It is noted that no displacement is detected for no gas flow rate (i.e., no plasma) even when the rf power is turned on. The absolute value of the force is obtained by multiplying the calibration coefficient relating the displacement to the force.

### Thrust balance calibration

After installing all the thruster components (including the source tube, the solenoid, the power cable for the solenoid, the gas injector, the antenna) and before pumping the vacuum chamber, a calibration procedure is performed by applying known axial forces to the balance and measuring the displacements. To apply the known axial force, known mass pieces are put on a calibration basket attached to a horizontal thread connected to the thruster, where a second thread is further attached to the support mounted on the chamber. When the angle $\theta$, the horizontal distance *L*, and the height *H*, are given as indicated in Fig. 9, the axial force $F_{cal}$ exerted to the thruster structure can be calculated from the force balance in a simple mechanics as

3 $$\begin{aligned} F_{cal} = mg \cot {\theta } = mg\frac{L}{H}. \end{aligned}$$

The distance *L* and the height *H* are measured by a straightedge with the error of $0.5\%$, and the mass of the piece measured by an electronic mass balance (with a resolution of 0.001 g) is $0.3234\pm 0.005$ g. The error $\sigma _{F}$ in the force can be estimated as $1.7\%$ from the propagation law of the error given by

4 $$\begin{aligned} \sigma _F= & {} \sqrt{\sigma _m^2 \left( \frac{\partial F_{cal}}{\partial m}\right) ^2 + \sigma _L^2 \left( \frac{\partial F_{cal}}{\partial L}\right) ^2 + \sigma _H^2 \left( \frac{\partial F_{cal}}{\partial H}\right) ^2}, \end{aligned}$$

where $\sigma _m$, $\sigma _L$, and $\sigma _H$ are the errors of *m*, *L*, and *H*, respectively.

**Figure 10:**
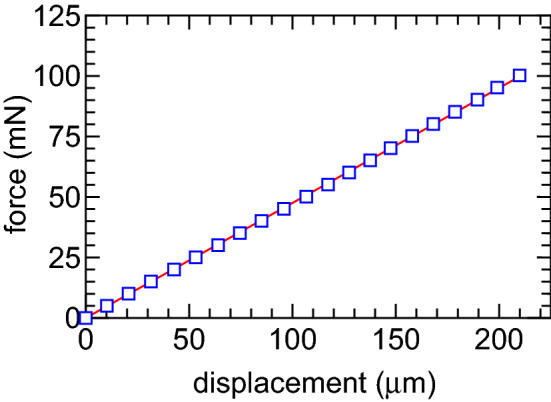
Calibration curve relating the displacement to the force, where the displacement is measured by the laser displacement sensor when applying known axial forces to the balance.

Figure 10 shows the typical relation between the measured displacement and the applied force (open squares). The characteristic can be fitted by a linear line as drawn by a solid line in Fig. 10; hence the linearly is well maintained over the force range including the thrust detected in the present experiment. The fitted line gives the calibration coefficient as $\sim 0.475\,\hbox {mN}/\upmu \hbox {m}$, which is confirmed to be unchanged after venting the chamber.

### rf power transfer efficiency

The rf power transfer efficiency $\eta _p$ is defined by a ratio of the rf power absorbed by the plasma to the net power from the rf generator. Assuming that the rf power is consumed by the plasma and the rf circuit including the antenna, the power transfer efficiency can be written as

5 $$\begin{aligned} \eta _p = \frac{R_p}{R_{total}} = \frac{R_{total}-R_{vac}}{R_{total}}, \end{aligned}$$

where $R_p$, $R_{vac}$, $R_{total}$ are the resistances of the plasma, the rf circuit, and the total load (the plasma and the circuit) during the plasma production. $R_{total}$ and $R_{vac}$ are estimated from the rf antenna current measured by a current probe (with an accuracy of $\pm \, 1\%$), and the net rf powers with the plasma and with no plasma (i.e., no gas), respectively, where the impedance matching circuit is tuned so as to minimize the reflected rf power. The method to obtain the rf power transfer efficiency has been widely used^66,67^ and has also shown good agreement with a theoretical model^68^. Since the rf antenna is water cooled to being maintained at a constant temperature, the resistance of the rf antenna is considered to be unchanged during the experiment.

### Langmuir probe

The Langmuir probe used in the present experiment has a radially facing 3-mm-diameter planar tip, where the one side of the tip surface is covered by a ceramic paste. A bipolar voltage power supply is connected to the probe via a resistor. By sweeping the bias voltage, the current-voltage characteristic can be obtained, where the current signal is obtained by measuring the voltage across the resistor via an precise isolation amplifier. The tangential line of the electron current in a semi-logarithm plot of the current-voltage characteristic can give an electron temperature. The measured electron temperature $T_e$ at $(r, z) = (0, 0)$ is $5.3\pm 0.5$ eV for the $m=+1$ mode antenna and $5.4\pm 0.6$ eV for the $m=0$ mode antenna. The plasma density $n_p$ can be estimated from the ion saturation current $I_{is}$ of the Langmuir probe, which is measured by biasing the probe to $-70$ V and given by

6 $$\begin{aligned} I_{is} = 0.61 e n_p u_B S, \end{aligned}$$

where *e*, $u_B$, and *S* are the elementary charge, the Bohm velocity given by $u_B = \sqrt{k_B T_e/m_i}$ with the Boltzmann constant $k_B$ and the ion mass $m_i$, and the collecting surface area of the Langmuir probe, respectively. By mounting the Langmuir probe on the movable stage installed inside the vacuum chamber, the two-dimensional profile of the ion saturation current $I_{is}$ can be measured.

## Data Availability

The data presented in this study are available from the author (K.T.) upon reasonable request.
